# Stage-of-Action Characterization of a Non-Sulfated Heteropolysaccharide from *Gracilaria lemaneiformis* Against Dengue Virus Serotype 2

**DOI:** 10.3390/v18060594

**Published:** 2026-05-24

**Authors:** Jiaxin Dai, Yingfang Liu, Jingshu Li, Zihan He, Kexin Xi, Yushan Jiang, Xuenan Zhang, Kefeng Wu, Bao Zhang, Wei Zhao, Weiwei Xiao

**Affiliations:** 1 School of Public Health, Guangdong Medical University, Dongguan 523808, China; dwinterelephant@outlook.com (J.D.); zihan_he@foxmail.com (Z.H.); 2 BSL-3 Laboratory, Guangdong Provincial Key Laboratory of Tropical Disease Research, Key Laboratory of Infectious Diseases Research in South China, School of Public Health, Southern Medical University, Guangzhou 510515, China; lyingfang22@163.com (Y.L.); jingshu@czc.hokudai.ac.jp (J.L.); xkexin317@163.com (K.X.); yushan.y.jiang@gmail.com (Y.J.); 15776619866@163.com (X.Z.); 3 The Marine Biomedical Research Institute of Guangdong Zhanjiang, School of Ocean and Tropical Medicine, Guangdong Medical University, Zhanjiang 524023, China

**Keywords:** dengue virus, antiviral polysaccharide, *Gracilaria lemaneiformis*, non-sulfated polysaccharide, time-of-addition, antiviral mechanism, progeny virus output

## Abstract

Marine algal polysaccharides have been widely investigated as antiviral candidates, yet nearly all anti-dengue studies have focused on sulfated species. Whether algal polysaccharides lacking prominent sulfation can inhibit dengue virus (DENV) remains unexplored. Here, we profiled the stage-specific antiviral activity of a heteropolysaccharide (GLHP) from *Gracilaria lemaneiformis*, whose Fourier-transform infrared (FT-IR) spectrum lacks characteristic sulfate ester absorption bands, against DENV serotype 2 (DENV-2) in Huh7 and BHK-21 cells. GLHP exhibited low cytotoxicity (CC_50_ exceeding 1000 μg/mL in Huh7 cells and approximately 950 μg/mL in BHK-21 cells). Time-of-addition analysis revealed that co-inoculation GLHP treatment (Co-inoc.) produced the strongest and most consistent inhibition of intracellular viral RNA, whereas pre-inoculation GLHP treatment (Pre-inoc.) was ineffective, indicating that the antiviral activity is predominantly associated with the virus–cell contact and entry stage. GLHP additionally reduced extracellular progeny virus output under post-inoculation GLHP treatment (Post-inoc.) conditions, and this reduction exceeded the corresponding change in intracellular viral RNA levels, suggesting an additional effect that may involve either a late replication step or secondary entry blockade of progeny virions. Attenuation of virus-induced cytopathic effects under Co-inoc. conditions further supported the antiviral activity. To our knowledge, these findings identify GLHP as the first non-sulfated marine polysaccharide shown to exhibit stage-defined antiviral activity against DENV-2 and support further investigation of its antiviral potential and structural determinants.

## 1. Introduction

Dengue virus (DENV), transmitted by *Aedes* mosquitoes, is one of the most widespread arboviral pathogens, with an estimated 390 million infections occurring annually, of which approximately 96 million are clinically apparent [1]. DENV is an enveloped, positive-sense single-stranded RNA virus of the family *Flaviviridae*. DENV belongs to the species *Orthoflavivirus denguei* and comprises four antigenically distinct serotypes (DENV-1 to -4), each capable of causing the full clinical spectrum. DENV-2 has been particularly associated with severe disease in secondary heterotypic infections through antibody-dependent enhancement and is among the most prevalent serotypes in hyperendemic regions [1,2]. The viral envelope glycoprotein E mediates receptor binding and membrane fusion during cell entry, and is the principal target of neutralizing antibodies [3]. Clinical outcomes range from asymptomatic infection to dengue hemorrhagic fever and shock syndrome [4]. The disease is endemic in more than 100 countries, predominantly in tropical and subtropical regions [5]. Two vaccines are currently licensed for dengue prevention: Dengvaxia^®^ (Sanofi Pasteur), restricted to seropositive individuals aged nine years and older, and Qdenga^®^ (Takeda), indicated for individuals aged four years and older regardless of prior dengue exposure status. Both are designed to protect against all four DENV serotypes [6,7]. No specific antiviral drug has been approved for clinical use, and treatment remains supportive [8,9]. Natural products have been explored as a source of candidate dengue antivirals, which include polyphenols, flavonoids, alkaloids, and marine polysaccharides [10].

Among natural products, polysaccharides from marine algae have attracted particular attention because of their structural diversity, broad spectrum of bioactivities, and favorable safety profiles [11,12]. Sulfated polysaccharides, including carrageenan and fucoidan, inhibit a range of enveloped viruses—herpes simplex virus, human immunodeficiency virus, and influenza virus—primarily by interfering with viral adsorption and entry [13]. Carrageenan inhibited DENV-2 at the adsorption and internalization stages in a serotype- and cell-type-dependent manner [14]. A polysaccharide from *Sargassum fusiforme* was reported to inhibit respiratory syncytial virus through a similar entry-blocking mechanism [15]. Fucoidan from *Sargassum* spp. also reduced DENV-2 infection by interfering with virus–cell binding [16]. Sulfation has therefore been widely regarded as a key structural determinant of antiviral activity in algal polysaccharides.

Within the dengue field, sulfated polysaccharides from red algae have been the most extensively studied. This activity has been attributed to electrostatic interactions between polyanionic sulfate groups and viral surface glycoproteins [13,17]. Across diverse algal sources, anti-DENV effectiveness has been shown to correlate with structural features such as sulfate content and molecular weight [18,19]. These studies have, however, focused almost exclusively on sulfated polysaccharides. Although a small number of non-sulfated polysaccharides, such as nostoflan from the cyanobacterium *Nostoc flagelliforme*, which inhibits herpes simplex virus and influenza virus entry [20], have demonstrated antiviral activity against other viruses; no non-sulfated algal polysaccharide has been tested against DENV.

*Gracilaria lemaneiformis* is a commercially important red alga widely cultivated along the Chinese coast. Polysaccharides from this species possess immunomodulatory [21], anti-inflammatory [22], and gut microbiota-regulating activities [23]. Feng et al. previously isolated and characterized a novel heteropolysaccharide (GLHP) from *G. lemaneiformis* [24]. GLHP has a molecular weight of 47,600 Da and is composed predominantly of galactose (62.08%) and glucosamine (14.72%) [24]. Notably, the Fourier-transform infrared (FT-IR) spectrum of GLHP showed no characteristic sulfate ester absorption bands [24], indicating that it is not a prominently sulfated polysaccharide. GLHP exhibits anti-pigmentation [24] and anti-photoaging activity [25], but its antiviral potential has not been investigated, and more broadly, whether algal polysaccharides lacking prominent sulfation can inhibit DENV remains untested. As a structurally characterized non-sulfated heteropolysaccharide with established biocompatibility, GLHP is a well-defined candidate to address this question; a positive result would extend the known antiviral repertoire of algal polysaccharides beyond the sulfated class.

In the present study, we evaluated the antiviral activity of GLHP against DENV-2 in two permissive cell lines: Huh7, a human hepatoma line reflecting the hepatic tropism of DENV, and BHK-21, a standard cell line widely used for DENV propagation and antiviral evaluation. Time-of-addition analysis was used to determine which stages of the viral life cycle are affected by GLHP. Extracellular progeny virus output and cytopathic effects were also assessed. Together, these experiments define the stage-specific antiviral profile of GLHP against DENV-2.

## 2. Materials and Methods

### 2.1. Preparation of GLHP

GLHP was isolated and characterized from *Gracilaria lemaneiformis* as previously described [24]. Lyophilized GLHP powder was kindly provided by Prof. Wu (Marine Biomedical Research Institution, Guangdong Medical University, Zhanjiang, China). The powder was dissolved in ultrapure water to prepare a stock solution (2 mg/mL), filter-sterilized (0.22 μm; Thermo Fisher Scientific, Waltham, MA, USA), and stored at −20 °C until use.

### 2.2. Cell Culture and Virus Propagation

Huh7 cells, BHK-21 cells, and DENV-2 (NGC strain) were obtained from the Guangdong Provincial Key Laboratory of Tropical Disease Research (School of Public Health, Southern Medical University, Guangzhou, China). All virus-related experiments were performed in the BSL-3 laboratory in compliance with applicable biosafety regulations. Huh7 cells were cultured in Dulbecco’s modified Eagle’s medium (DMEM) and BHK-21 cells in RPMI 1640 medium, each supplemented with 10% fetal bovine serum (FBS; Sangon Biotech, Shanghai, China), at 37 °C with 5% CO_2_. DENV-2 was propagated in C6/36 cells, and viral titers were determined by plaque assay in BHK-21 cells as previously described [26]. Aliquots were stored at −80 °C until use. Maintenance medium containing 2% FBS was used for compound dilution and incubation during treatment periods.

### 2.3. Cell Viability Assay

Cytotoxicity was evaluated using the CCK-8 assay (Solarbio, Beijing, China). Cells were seeded in 96-well plates at a density of 1 × 10^4^ cells per well and incubated overnight. Cells were treated with GLHP at the indicated concentrations or ribavirin (Renhe Pharmaceutical, Zhangshu, China) for 48 h. CCK-8 reagent was added according to the manufacturer’s instructions, and absorbance was measured at 450 nm. Cell viability was expressed as a percentage relative to the untreated control. The 50% cytotoxic concentration (CC_50_) was calculated from the dose–response data.

### 2.4. Time-of-Addition Antiviral Assays

Huh7 and BHK-21 cells were seeded in 24-well plates at 5 × 10^4^ cells per well and incubated overnight. Cells were infected with DENV-2 in serum-free medium at a multiplicity of infection (MOI) of 1 (Huh7) or 0.001 (BHK-21) for 2 h at 37 °C. The MOI for each cell line was selected on the basis of preliminary experiments to achieve consistent infection levels suitable for quantitative assessment within the 48 h observation period, reflecting differences in the permissiveness of the two cell lines to DENV-2. In all assays, the virus control group received an equivalent volume of vehicle (ultrapure water in maintenance medium) in place of GLHP.

In the all-treatment protocol, cells were pre-incubated with GLHP (125, 250, or 500 μg/mL) for 2 h before infection. GLHP remained present during adsorption and was reapplied in maintenance medium after removal of unbound virus. Cells were harvested at 48 hpi.

In the pre-inoculation GLHP treatment (Pre-inoc.) protocol, cells were incubated with GLHP for 2 h before infection. GLHP was removed before adsorption. After removal of unbound virus, cells were maintained in GLHP-free medium and harvested at 48 hpi.

In the co-inoculation GLHP treatment (Co-inoc.) protocol, GLHP and DENV-2 were mixed (1:1, *v*/*v*) and added to cells for 2 h. After removal of the mixture, cells were maintained in GLHP-free medium and harvested at 48 hpi.

In the post-inoculation GLHP treatment (Post-inoc.) protocol, cells were infected for 2 h. After removal of unbound virus, GLHP-containing medium was added until harvest at 48 hpi.

Ribavirin (0.4 μg/mL) was included as a reference compound in the all-treatment, Pre-inoc., and Co-inoc. assays. Ribavirin was not included in the Post-inoc. assay because the primary objective of this study was to characterize the stage-specific activity of GLHP rather than to benchmark against a replication inhibitor at each stage. Intracellular viral RNA was quantified by RT-qPCR as described in Section 2.6.

### 2.5. Extracellular Progeny Virus Quantification

Culture supernatants were collected from DENV-2-infected BHK-21 cells at 48 hpi under Pre-inoc., Co-inoc., and Post-inoc. conditions as described in Section 2.4. Viral RNA was extracted from supernatants using the NucleoSpin RNA Virus Kit (Macherey-Nagel, Düren, Germany) and quantified by absolute RT-qPCR as described in Section 2.6. Results were expressed as log DENV-2 copies/mL.

### 2.6. RNA Extraction and RT-qPCR

Intracellular total RNA was extracted using the SteadyPure Universal RNA Extraction Kit II (Accurate Biotechnology, Changsha, China). Reverse transcription was performed using the Evo M-MLV RT Premix for qPCR (Accurate Biotechnology, Changsha, China).

For absolute quantification, qPCR was performed using the Pro Taq HS Premix Probe qPCR Kit II (Accurate Biotechnology, Changsha, China) with a DENV-2-specific primer–probe set: forward, 5′-CAGTCGGAAATGACACAG-3′; reverse, 5′-GCAACACCATCTCATTGA-3′; the TaqMan probe was a dual-labeled oligonucleotide with a 5′-FAM fluorophore and 3′-BHQ1 quencher, 5′-FAM-AAGTAACACCACAGAGTTCCATCACA-BHQ1-3′. A standard curve was generated using serial dilutions of a DENV-2 plasmid standard. The plasmid was constructed by OncoImmune (Guangzhou, China) and its concentration was provided. All RT-qPCR analyses used absolute quantification unless otherwise noted.

Relative quantification was adopted for the Co-inoc. assay in Huh7 cells because the viral RNA levels in this experimental group fell outside the reliable range of the absolute standard curve; relative quantification against GAPDH provided more robust normalization under these conditions. This was performed using the SYBR Green Premix Pro Taq HS qPCR Kit II (Accurate Biotechnology, Changsha, China) with DENV-2-specific primers (forward, 5′-CAGGCTATGGCACTGTCACGAT-3′; reverse, 5′-CCATTTGCAGCAACACCATCTC-3′) and human GAPDH as the internal reference (forward, 5′-CATCCTGGGCTACACTGAGC-3′; reverse, 5′-AAAGTGGTCGTTGAGGGCAA-3′). Viral RNA levels were normalized to GAPDH and expressed relative to the virus control.

### 2.7. Cytopathic Effect Observation

Morphological changes in Huh7 and BHK-21 cells under Co-inoc. conditions were examined under phase-contrast illumination using an inverted microscope (Nikon Eclipse Ti2, Tokyo, Japan) at ×125 and ×400 magnification.

### 2.8. Statistical Analysis

Data are presented as mean ± SD. The data were tested for normality and homogeneity of variance. Statistical significance was assessed by one-way ANOVA followed by Dunnett’s post hoc test. *p* < 0.05 was considered statistically significant. All analyses were performed using GraphPad Prism 10.0 (GraphPad Software, San Diego, CA, USA).

## 3. Results

### 3.1. GLHP Exhibits Low Cytotoxicity and Demonstrates Antiviral Activity Against DENV-2 In Vitro

**Figure 1:**
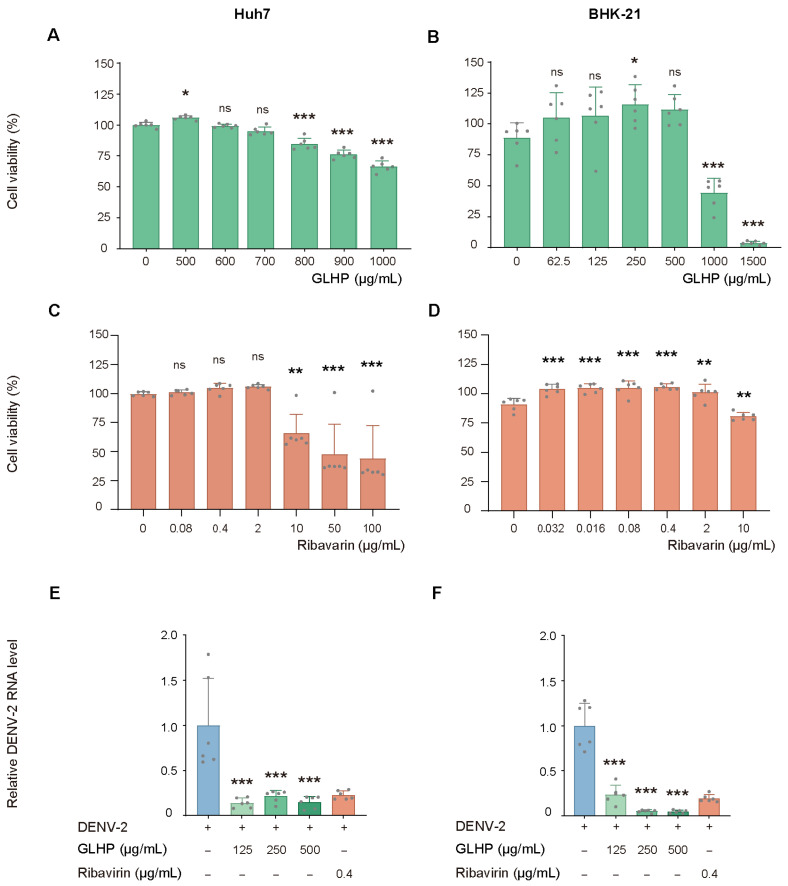
Cytotoxicity of GLHP and ribavirin, and antiviral activity of GLHP against DENV-2 under all-treatment conditions. (**A**) Cytotoxicity of GLHP in Huh7 cells. (**B**) Cytotoxicity of GLHP in BHK-21 cells. (**C**) Cytotoxicity of ribavirin in Huh7 cells. (**D**) Cytotoxicity of ribavirin in BHK-21 cells. (**E**) Relative DENV-2 RNA levels in Huh7 cells under all-treatment conditions. (**F**) Relative DENV-2 RNA levels in BHK-21 cells under all-treatment conditions. Cell viability was determined by CCK-8 assay. Viral RNA levels were quantified by RT-qPCR and normalized to the virus control. Ribavirin (0.4 μg/mL) was included as a reference compound in (**E**,**F**). In (**A**,**B**), bar color green represent GLHP. In (C,D), bar color orange represent ribavirin. In (**E**,**F**), bar colors represent: blue, DENV-2 group; light green, GLHP 125 μg/mL; medium green, GLHP 250 μg/mL; dark green, GLHP 500 μg/mL; orange, ribavirin 0.4 μg/mL. Grey dots represent individual data points. Data are presented as mean ± SD. In (**A**–**D**), significance was assessed versus the 0 μg/mL group. In (**E**,**F**), significance was assessed versus the virus control. ns, not significant; * *p* < 0.05, ** *p* < 0.01, *** *p* < 0.001. Cell viability values slightly exceeding 100% at lower GLHP concentrations are consistent with the mild proliferative activity reported for *G. lemaneiformis* polysaccharides at sub-cytotoxic concentrations [21], and do not indicate cytotoxicity.

We first evaluated the cytotoxicity of GLHP and ribavirin in Huh7 and BHK-21 cells by the CCK-8 assay (Figure 1A–D). GLHP showed low cytotoxicity in both cell lines, with a CC_50_ exceeding 1000 μg/mL in Huh7 cells and approximately 950 μg/mL in BHK-21 cells. Concentrations used in subsequent antiviral experiments (125–500 μg/mL) fell well below these values. For ribavirin, the CC_50_ was estimated to be between 10 and 50 μg/mL in Huh7 cells based on the dose–response profile and >10 μg/mL in BHK-21 cells; the working concentration (0.4 μg/mL) was well below these thresholds.

We then assessed the antiviral activity of GLHP under all-treatment conditions, in which GLHP was present throughout the experiment (Figure 1E,F). GLHP reduced DENV-2 RNA levels at all tested concentrations in both cell lines relative to the virus control. Ribavirin (0.4 μg/mL), included as a reference compound, also reduced viral RNA levels in both cell lines. In BHK-21 cells, the inhibitory effect showed a concentration-dependent trend, whereas in Huh7 cells, the effect was pronounced at the lowest concentration tested (125 μg/mL). These results confirmed the antiviral activity of GLHP against DENV-2 in vitro and provided the rationale for subsequent time-of-addition experiments.

### 3.2. Time-of-Addition Analysis Indicates That GLHP Predominantly Affects Early Infection Events

**Figure 2:**
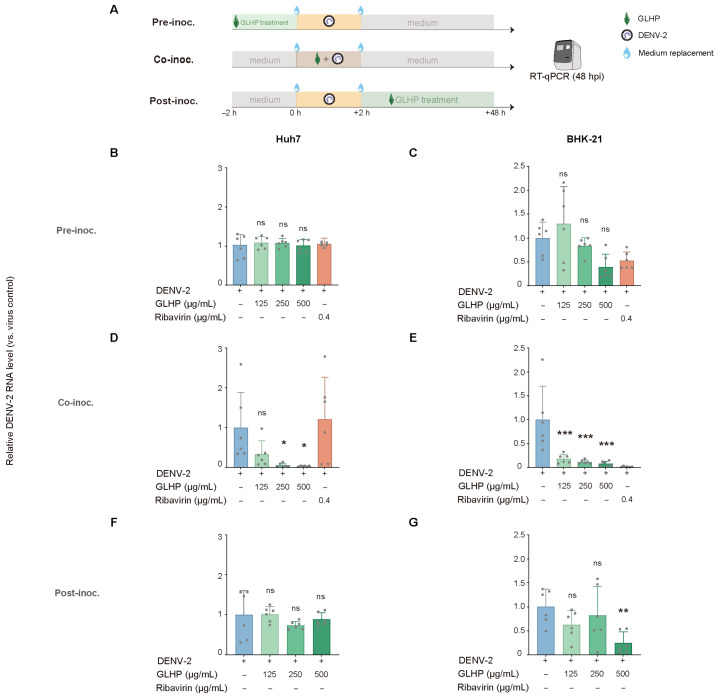
Time-of-addition analysis of the antiviral activity of GLHP against DENV-2. (**A**) Schematic of the pre-inoculation GLHP treatment (Pre-inoc.), co-inoculation GLHP treatment (Co-inoc.), and post-inoculation GLHP treatment (Post-inoc.) protocols. Time markers indicate −2 h (GLHP treatment or medium treatment), 0 h (DENV-2 inoculation), +2 h (removal of supernatant and wash, replacement of GLHP treatment or medium treatment), and +48 h (harvest). Blue droplet symbols indicate medium replacement, green seaweed symbols indicate GLHP, and viral particle symbols indicate DENV-2. (**B**) Relative DENV-2 RNA levels in Huh7 cells under Pre-inoc. conditions. (**C**) Relative DENV-2 RNA levels in BHK-21 cells under Pre-inoc. conditions. (**D**) Relative DENV-2 RNA levels in Huh7 cells under Co-inoc. conditions. (**E**) Relative DENV-2 RNA levels in BHK-21 cells under Co-inoc. conditions. (**F**) Relative DENV-2 RNA levels in Huh7 cells under Post-inoc. conditions. (**G**) Relative DENV-2 RNA levels in BHK-21 cells under Post-inoc. conditions. Viral RNA levels were quantified by RT-qPCR and normalized to the corresponding virus control. In (**B**–**G**), bar colors represent: blue, DENV-2 group; light green, GLHP 125 μg/mL; medium green, GLHP 250 μg/mL; dark green, GLHP 500 μg/mL; orange, ribavirin 0.4 μg/mL. (**B**–**G**) share the same *y*-axis range. Grey dots represent individual data points. Data are presented as mean ± SD. ns, not significant; * *p* < 0.05, ** *p* < 0.01, *** *p* < 0.001 versus the corresponding virus control.

To determine which stage of the viral life cycle GLHP affects, we performed a time-of-addition analysis using three treatment protocols: Pre-inoc., Co-inoc., and Post-inoc. (Figure 2A).

Under Pre-inoc. conditions, GLHP did not significantly reduce DENV-2 RNA levels at any tested concentration in either Huh7 or BHK-21 cells (Figure 2B,C).

Co-inoc. produced the strongest and most consistent inhibition across both cell lines. In Huh7 cells, GLHP reduced DENV-2 RNA levels at 250 and 500 μg/mL (Figure 2D). In BHK-21 cells, GLHP reduced DENV-2 RNA levels at all three concentrations (Figure 2E). Inhibition increased with GLHP concentration in both cell lines.

Under Post-inoc. conditions, GLHP did not significantly reduce DENV-2 RNA levels in Huh7 cells at any concentration (Figure 2F). In BHK-21 cells, GLHP reduced DENV-2 RNA levels only at the highest concentration (Figure 2G). The differential Post-inoc. response between the two cell lines may reflect the substantially higher MOI used for Huh7 cells (1 vs. 0.001 for BHK-21), which could saturate intracellular replication and narrow the window for post-entry intervention. Together, these results indicate that GLHP predominantly affects early infection events during virus–cell contact and entry. Immunofluorescence imaging of the DENV-2 E protein under Co-inoc. conditions provided additional visual support for the Co-inoc. effect in both cell lines (Supplementary Figure S1).

### 3.3. GLHP Additionally Affects Progeny Virus Production and Attenuates Cytopathic Effects in DENV-2-Infected Cells

We next examined the effects of GLHP on progeny virus production and virus-induced cytopathic changes.

**Figure 3:**
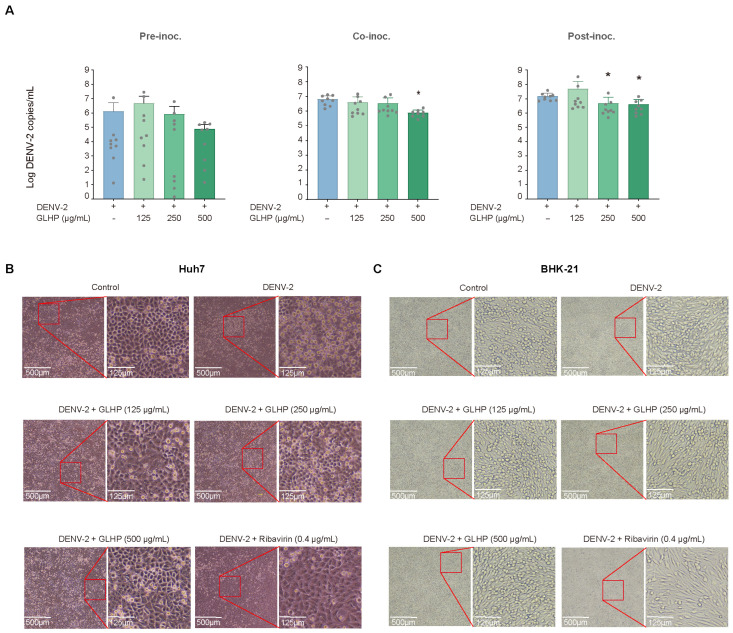
Effect of GLHP on extracellular progeny virus output and cytopathic effect in DENV-2-infected cells. (**A**) Extracellular progeny virus output in culture supernatants of BHK-21 cells under Pre-inoc., Co-inoc., and Post-inoc. conditions, quantified by RT-qPCR and expressed as log DENV-2 copies/mL. Bar colors represent: blue, DENV-2 group; light green, GLHP 125 μg/mL; medium green, GLHP 250 μg/mL; dark green, GLHP 500 μg/mL. Grey dots represent individual data points. (**B**) Representative CPE images of Huh7 cells in the Co-inoc. assay. (**C**) Representative CPE images of BHK-21 cells in the Co-inoc. assay. Red boxes indicate regions shown at higher magnification. Scale bars: 500 μm (original images); 125 μm (enlarged images). Data in (**A**) are presented as mean ± SD. * *p* < 0.05 versus the corresponding virus control.

We quantified extracellular progeny virus output in culture supernatants from DENV-2-infected BHK-21 cells (Figure 3A). GLHP did not reduce extracellular progeny virus output under Pre-inoc. conditions. Under Co-inoc. conditions, GLHP reduced progeny virus output only at 500 μg/mL. Under Post-inoc. conditions, GLHP reduced progeny virus output at 250 and 500 μg/mL. Notably, the reduction in progeny virus output under Post-inoc. conditions was more pronounced than the corresponding change in intracellular viral RNA levels (Figure 2F,G), suggesting that the additional effect of GLHP may extend beyond genome replication.

**Figure 4:**
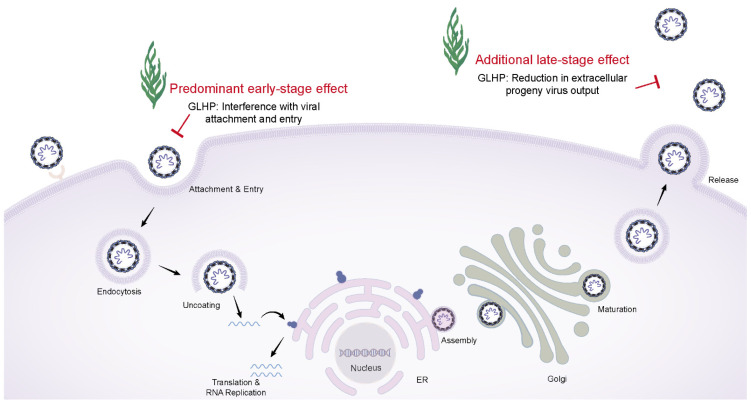
Working model of the stage-specific antiviral activity of GLHP against DENV-2. GLHP activity is predominantly associated with the virus–cell contact and entry stage (Co-inoc.). The reduction in extracellular progeny virus output under Post-inoc. conditions may reflect either a late-stage effect or secondary entry blockade of released progeny virions; the specific mechanism remains to be determined. The green seaweed icons denote GLHP. Spherical particles with surface projections represent DENV-2 virions. Red blunt-ended arrows indicate inhibition by GLHP. Black arrows indicate the sequential steps of the DENV-2 replication cycle. Wavy lines represent viral RNA. The pink tubular network depicts the endoplasmic reticulum (ER), and the studded structures on the ER membrane represent membrane-bound ribosomes. The olive-green stacked cisternae depict the Golgi apparatus.

We examined virus-induced morphological changes in Huh7 and BHK-21 cells under Co-inoc. conditions (Figure 3B,C). DENV-2 infection induced evident cytopathic changes in both cell lines compared with uninfected control cells. GLHP attenuated these changes in both Huh7 and BHK-21 cells. This protective effect was more pronounced at higher concentrations. These morphological findings were consistent with the Co-inoc. RT-qPCR results. Together, these results indicate that GLHP additionally affects progeny virus production and attenuates virus-induced cytopathic effects. The specific late-stage step associated with this additional antiviral effect remains undefined. A working model summarizing the stage-specific antiviral profile of GLHP is presented in Figure 4.

## 4. Discussion

The present study characterized the stage-of-action profile of GLHP against DENV-2 using multiple independent experimental endpoints in two permissive cell lines of different species origin. Time-of-addition analysis demonstrated that Co-inoc.—in which GLHP is present during the virus–cell contact and entry window but absent thereafter—produced the strongest and most consistent inhibition in both Huh7 and BHK-21 cells, whereas Pre-inoc. produced no statistically significant effect. Post-inoc. conditions reduced extracellular progeny viral output in BHK-21 cells. Together with the attenuation of cytopathic effects and reduced E protein immunofluorescence under Co-inoc. conditions (Supplementary Figure S1), these findings converge on a coherent antiviral profile in which GLHP predominantly acts at the virus–cell contact and entry stage. To our knowledge, this is the first stage-of-action characterization of a non-sulfated algal polysaccharide (as defined by the absence of sulfate ester absorption bands in FT-IR spectroscopy [24]) against DENV-2. A working model summarizing this dual-stage antiviral profile is shown in Figure 4.

GLHP exhibited low cytotoxicity in both Huh7 and BHK-21 cells, with CC_50_ values that provided an adequate experimental window for antiviral evaluation. Because viral RNA inhibition already exceeded 60% at the lowest tested concentration (125 μg/mL), a conventional IC_50_ could not be determined from the current dose range. The concentration range (125–500 μg/mL) was selected on the basis of preliminary cytotoxicity data and prior bioactivity studies of GLHP; lower concentrations were not included because GLHP showed only marginal inhibition below 125 μg/mL in earlier screening, making this range insufficient for reliable dose–response fitting. Extending the assay to a finer low-dose series in future work would precisely determine the IC_50_ and strengthen dose–response characterization. Selectivity indices were therefore estimated conservatively using 125 μg/mL as a surrogate upper bound for IC_50_, yielding SI > 8 (Huh7) and SI ≈ 7.6 (BHK-21); the true selectivity indices are likely substantially higher. This favorable safety margin is consistent with the broader biocompatibility of natural polysaccharides [11,27]. The consistent antiviral activity observed under all-treatment conditions across both cell lines further supports the robustness of the GLHP antiviral effect. The concordance of the Co-inoc. effect across a human hepatoma line (Huh7) and a hamster kidney line (BHK-21)—which differ in species origin, tissue type, and receptor repertoire—suggests that the antiviral activity of GLHP is not restricted to a single cellular context.

Early-stage interference is the dominant component of GLHP activity. Pre-inoc. alone produced no significant antiviral effect, although a numerical downward trend was observed in some groups; this trend may reflect residual surface-associated GLHP that resists removal by standard washing, given the known capacity of polysaccharides to interact non-covalently with cell-surface glycans. Co-inoc. yielded the strongest and most consistent inhibition in both cell lines, indicating that GLHP acts mainly during virus–cell contact and entry. A similar stage preference has been reported for sulfated polysaccharides: Talarico et al. identified adsorption and internalization as the principal steps affected by carrageenan [14,17] and Ichiyama et al. reported that curdlan sulfate inhibited DENV at the binding and early post-attachment stages [28]. The concordance of the Co-inoc. effect across Huh7 and BHK-21 cells—which differ in species origin (human versus hamster), tissue type (hepatoma versus kidney), and receptor repertoire—suggests that the antiviral activity is not restricted to a single cellular context. Sub-log reductions in intracellular viral RNA, observed in some groups, are characteristic of polysaccharide entry inhibitors that act stoichiometrically on virion–cell interaction rather than catalytically on intracellular replication, and are consistent with effect magnitudes reported for carrageenan and fucoidan in this literature [16,17].

The activity of these sulfated polysaccharides has been attributed to sulfate–glycoprotein electrostatic interactions [18,29]. However, the FT-IR spectrum of GLHP lacks the characteristic sulfate ester absorption bands (typically near 1240 cm^−1^ for S=O asymmetric stretching and 820–850 cm^−1^ for C–O–S bending) [24], which argues against a classical polyanion-mediated mechanism. Nonetheless, the absence of these prominent spectral features distinguishes GLHP from the sulfated polysaccharides that have dominated anti-DENV research to date. Notably, DENV entry involves not only sulfated GAGs but also non-sulfated carbohydrate co-receptors; the Galβ1-4GlcNAc motif of neolactotetraosylceramide (nLc4Cer) has been identified as a critical determinant for DENV-2 binding on BHK-21 cells [30]. Given that galactose and glucosamine are the two most abundant monosaccharides in GLHP [24], competitive interference with such carbohydrate-mediated virus–cell interactions represents a plausible sulfation-independent mechanism that warrants direct investigation. The persistence of inhibition after GLHP removal further argues against purely reversible competitive displacement, suggesting a durable surface interaction. The antiviral activity of a non-sulfated polysaccharide is noteworthy given that sulfation has generally been considered essential for polysaccharide-mediated viral inhibition. Non-sulfated polysaccharides were reported to lack anti-SARS-CoV-2 activity, in contrast to their sulfated counterparts [31], reinforcing the prevailing view that sulfation is a prerequisite. The present finding that GLHP inhibits DENV-2 despite the absence of detectable sulfation suggests that alternative, sulfation-independent mechanisms may operate in a virus-specific manner.

The reduction in extracellular progeny viral output under Post-inoc. conditions in BHK-21 cells admits two interpretations. The first is an effect on a late stage of the viral life cycle—assembly, maturation, or egress. GLHP did not proportionately reduce intracellular viral RNA levels, indicating that genome amplification itself was not the primary target; yet extracellular progeny virus output was significantly reduced, creating a clear dissociation between these two read-outs. This dissociation narrows the candidate steps to those occurring after genome replication, including virion assembly, maturation, egress, or a reduction in the specific infectivity of released particles [32]. A partial parallel exists with 9N-methylharmine, which similarly did not suppress viral RNA synthesis yet reduced infectious progeny output [33]. The second, more parsimonious interpretation is a secondary entry-blocking effect: in the Post-inoc. protocol, GLHP is continuously present in the medium from 2 h post-inoculation and therefore coexists with progeny virions as they are released; if GLHP engages these virions through the same mechanism proposed for the Co-inoc. effect, it would reduce secondary infection of neighboring cells. At the low MOI used for BHK-21 cells, the 48 h endpoint captures multi-cycle spread; suppressing secondary entry would therefore reduce cumulative extracellular viral RNA without proportionately affecting the initially infected cells. This interpretation unifies the antiviral observations under a single entry-stage mechanism. The two interpretations are experimentally distinguishable and represent immediate priorities for follow-up investigation. The higher MOI used for Huh7 cells (1 vs. 0.001) may saturate replication and narrow the window for detecting either post-entry or secondary-spread effects, potentially explaining the lack of a Post-inoc. effect in this cell line.

The use of Huh7 cells strengthens the translational relevance of these findings. The liver is a principal target organ of DENV infection, and hepatocyte injury is a hallmark of severe dengue disease [34]; demonstrating GLHP activity in a human hepatoma line connects the in vitro antiviral profile to a clinically relevant cellular context. Given that no antiviral therapy for dengue has been approved to date [8], compounds with defined stage-of-action profiles are needed to guide rational antiviral development. The predominant early-stage effect of GLHP directs attention toward virus–cell interaction mechanisms, while the additional late-stage effect identifies extracellular progeny virus output as a functional read-out for dissecting post-replication steps. The detailed structural characterization of GLHP [24]—including monosaccharide composition, glycosidic linkages, and molecular weight—enables targeted modifications such as selective sulfation in future structure–activity studies. *G. lemaneiformis* is a commercially cultivated red alga with a sustainable and scalable supply, which further supports the practical feasibility of such follow-up investigation. The high selectivity indices indicate that effective antiviral concentrations are well separated from cytotoxic thresholds, leaving room for dose optimization in future studies. Several limitations should be noted: the present study was conducted in vitro, and only DENV-2 was tested. Direct binding assays, pseudovirus-based entry experiments, plaque reduction assays, and head-to-head comparison with sulfated polysaccharides are priorities for the future follow-up mechanistic studies.

## 5. Conclusions

Taken together, our findings establish GLHP as, to our knowledge, the first non-sulfated algal polysaccharide shown to inhibit DENV-2 at defined infection stages, extending the known structural repertoire of anti-DENV algal polysaccharides beyond the sulfated class. These results provide a stage-of-action foundation for further mechanistic and structure–activity investigation.

## Data Availability

The original contributions presented in this study are included in the article/Supplementary Materials. Further inquiries can be directed to the corresponding authors.

## Supplementary Materials

The following supporting information can be downloaded at: https://www.mdpi.com/article/10.3390/v18060594/s1, Figure S1: Immunofluorescence detection of DENV-2 E protein under co-inoculation GLHP treatment (Co-inoc.) conditions. Cells were co-treated with GLHP (500 μg/mL) or ribavirin and infected with DENV-2 simultaneously, then fixed and stained at 48 hpi. DENV-2 E protein was detected using the 4G2 monoclonal antibody with FITC-conjugated secondary antibody (green); nuclei were counterstained with DAPI (blue). (A) Huh7 cells: virus control and GLHP-treated groups. (B) BHK-21 cells: virus control, GLHP-treated, and ribavirin-treated groups. Scale bar, 100 μm. Mock-infected cells showed no detectable E protein signal under identical staining conditions.
