# Supplementary material for: Stage-of-Action Characterization of a Non-Sulfated Heteropolysaccharide from Gracilaria lemaneiformis Against Dengue Virus Serotype 2
Source: Viruses. 2026 May 24;18(6):594. doi: 10.3390/v18060594 (PMC13307904; doi:10.3390/v18060594)
Supplement: Supplementary file 1 [file viruses-18-00594-s001.zip › viruses-4293200-supplementary.pdf]

Supplementary Methods — Immunofluorescence assay

Cells cultured on coverslips were fixed with 4% paraformaldehyde at room temperature for 20 min, then permeabilized with 0.2% Triton X-100 at room temperature for 20 min. After blocking with 5% bovine serum albumin (BSA) in PBS for 1 h, cells were incubated overnight at 4°C with anti-DENV-2 E protein monoclonal antibody 4G2 (available in our laboratory). Following three washes with PBS, cells were incubated with FITC-conjugated goat anti-mouse IgG (H+L) (Proteintech, Wuhan, China) for 1 h at room temperature in the dark. Coverslips were then mounted with DAPI-containing anti-fade mounting medium. Images were acquired using a fluorescence microscope.

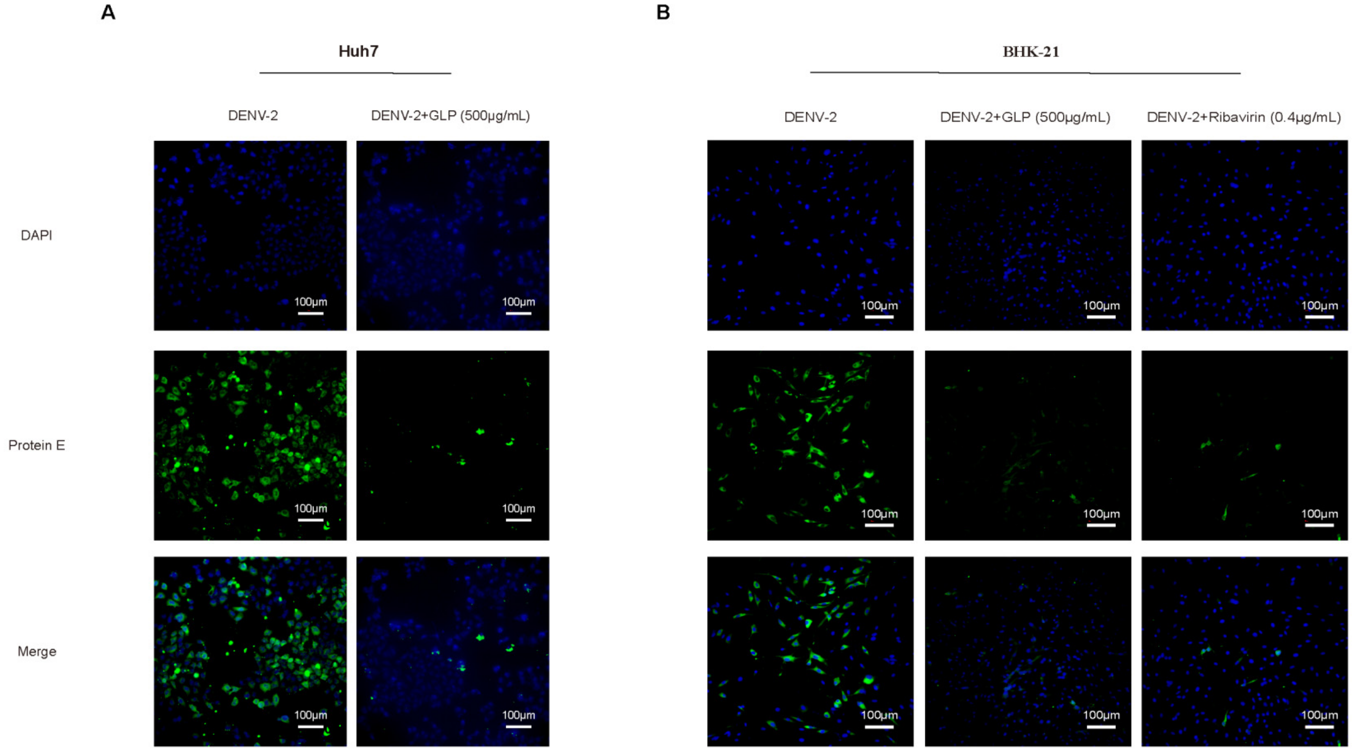

Figure S1. Immunofluorescence detection of DENV-2 E protein under co-inoculation GLHP treatment (Co-inoc.) conditions. Cells were co-treated with GLHP (500 μg/mL) or ribavirin and infected with DENV-2 simultaneously, then fixed and stained at 48 hpi. DENV-2 E protein was detected using the 4G2 monoclonal antibody with FITC-conjugated secondary antibody (green); nuclei were counterstained with DAPI (blue). (A) Huh7 cells: virus control and GLHP-treated groups. (B) BHK-21 cells: virus control, GLHP-treated, and ribavirin-treated groups. Scale bar, 100 μm. Mock-infected cells showed no detectable E protein signal under identical staining conditions.
